# Clinical factors linked to xylazine exposure in emergency department patients with illicit opioid overdose

**DOI:** 10.1111/add.70289

**Published:** 2026-01-13

**Authors:** Jennifer S. Love, Carmen Vargas-Torres, Kim Aldy, Jeffrey Brent, Paul Wax, Rachel Culbreth, Alex Krotulski, Sharan Campleman, Barry Logan, Stephanie Abston, Shao Li, Alex F. Manini

**Affiliations:** 1 Icahn School of Medicine at Mount Sinai, NYC Health + Hospitals/Elmhurst, Center for Research on Emerging Substances, Poisoning, Overdose and New Discoveries (RESPOND), New York, NY, USA; 2 American College of Medical Toxicology, Phoenix, AZ, USA; 3 University of Colorado School of Medicine, Aurora, CO, USA; 4 Center for Forensic Science Research and Education at the Fredric Rieders Family Foundation, Horsham, PA, USA; 5 NMS Labs, Horsham, PA, USA

**Keywords:** adulterants, bradycardia, emergency, fentanyl, opioids, overdose, xylazine

## Abstract

**Background and aims:**

Xylazine, an alpha-2 agonist used in veterinary anesthesia, is increasingly detected in the illicit opioid supply but little is known about the patient level factors associated with xylazine in non-fatal opioid overdose. This study aimed to determine the demographic and clinical factors associated with xylazine detection among emergency department (ED) patients with opioid overdose.

**Design:**

Observational study. The Toxicology Investigators Consortium (ToxIC) Fentalog Study is a multicenter, prospective cohort of adult patients with suspected opioid overdose. This analysis included patients enrolled from September 2020 to September 2023.

**Setting:**

In this multicenter study, participating sites included 10 institutions across 9 states in 4 regions of the United States (US): Northeast, Southeast, Midwest and West.

**Participants:**

Patients were eligible for Fentalog Study inclusion if they were at least 18 years old, had a suspected opioid overdose and had waste blood available for toxicologic analysis. Only patients with qualitative serum detection of illicit opioids and/or xylazine were included in the final cohort. Of 5554 patients screened, 1289 were eligible for Fentalog Study inclusion.

**Measurements:**

Based on results of liquid chromatography with a quadrupole time-of-flight mass spectrometer (LCQTOF-MS) and/or liquid chromatography with a triple quadrupole mass spectrometer (LC-QQQ-MS), patients were categorized into those with xylazine detected (positive cases) and without xylazine detected (negative controls). To determine clinical variables associated with xylazine detection, the primary outcome of interest was qualitative detection of xylazine on serum sampling by LCQTOF-MS.

**Findings:**

Xylazine was detected in 238 patients. Patients with xylazine were primarily male (78%), white (48%), non-Hispanic (82%) and located in the Northeast US (75%). Bradycardia on initial ED vital signs was associated with higher likelihood of xylazine detection (adjusted odds ratio = 2.11, 95% confidence interval = 1.06–4.06).

**Conclusions:**

Xylazine detection among emergency department opioid overdose patients appears to be more prevalent in the Northeast US and bradycardia appears to be a statistically significant clinical predictor.

## INTRODUCTION

In April 2023, xylazine-adulterated fentanyl and its health effects were declared a national toxicologic emergency by the US federal government [1]. By July 2023, a national response plan was published to study xylazine and its clinical effects on people who use drugs (PWUD) [2, 3]. Clinicians, policymakers and harm reduction experts have emphasized the need to establish robust evidence-based research to understand the pharmacology, clinical effects and potential treatments for xylazine [4, 5].

Xylazine, a veterinary medication in the alpha-2 agonist drug class, is structurally similar to clonidine and dexmedetomidine. It is not approved for human use but is a common sedative and pain medication in large animal anesthesia [6]. The alpha-2 agonist effects of xylazine in single-agent overdose include central nervous system (CNS) depressant effects and cardiovascular effects, such as bradycardia, hypotension and cardiac arrest.

The emergence of xylazine as a recreational drug supply adulterant, especially in non-prescription opioid drug supply, began in the early 2000s. Xylazine adulteration was first described among Puerto Rican PWUD, with common effects including sedation and skin wounds [7, 8]. Other effects of xylazine use include sedation, severe skin wounds and altered duration of euphoria [9–11]. Xylazine has been increasingly detected in source samples, including post-mortem serum, drug products and syringes, and almost always co-occurring with fentanyl [12–14]. Studies from Northeast and Mid-Atlantic states describe a rising burden of overdose deaths with xylazine [15–17] and increasing xylazine detection in drug-checking programs [13].

Limited knowledge exists of the toxicologic and addiction health effects of xylazine. Few survey studies have reported on demographic or clinical risk factors associated with xylazine-adulterated fentanyl [18, 19]. In this study, we identified demographic and clinical factors associated with xylazine detection among emergency department (ED) patients with analytically confirmed opioid overdose. We hypothesized that bradycardia and hypotension would be key clinical predictors given the classification of xylazine as an alpha-2 agonist drug.

## METHODS

### Design

The Toxicology Investigators Consortium (ToxIC) Fentalog Study is an ongoing, multi-center, prospective cohort of adult patients (aged 18-+ years) presenting to the ED with suspected opioid overdose. This study included patients who presented to one of 18 EDs at 10 participating healthcare institutions within the USA between September 2020 and September 2023. The EDs were from nine states: California, Colorado, Georgia, Michigan, Missouri, New York, New Jersey, Oregon and Pennsylvania. A central institutional review board (Western Copernicus Group IRB) provided approval with a waiver of informed consent, and each participating institution obtained local IRB approval.

### Participants

#### Inclusion/exclusion criteria

Patients aged 18 years or older who presented to the ED with a suspected opioid overdose from September 2020 to September 2023 were screened for Fentalog Study eligibility. Inclusion criteria were: (i) suspected opioid overdose, based on clinical presentation, chief complaint or discharge diagnosis; (ii) naloxone administration for overdose prior to arrival at the hospital and/or in the ED; or (iii) self-reported opioid use resulting in an ED visit for overdose. Patients who presented with trauma, were imprisoned persons or did not have waste blood, defined as blood collected during ED clinical care and left over after the ED visit, were excluded. Of those eligible for inclusion, patients with illicit opioids and/or xylazine detected on subsequent serum testing were included in this cohort. Because previous studies have consistently reported xylazine detection in combination with an illicit opioid, xylazine-positive cases without an opioid detected were included in the cohort.

#### Definitions

An illicit opioid was defined as heroin, non-prescription fentanyl, fentanyl analogs, nitazene analogs, brorphine or other new synthetic opioids. Patients with prescription fentanyl or other opioids (e.g. oxycodone, methadone) without xylazine detected were not included in the cohort.

Initial ED vital signs were dichotomized to define clinical parameters. Tachycardia was defined as an initial heart rate greater than 120 beats per minute (bpm). Bradycardia was defined as an initial heart rate less than 60 bpm. Hypertension was defined as an initial systolic blood pressure greater than or equal to 200 mmHg. Hypotension was defined as an initial systolic blood pressure less than or equal to 80 mmHg. Bradypnea was defined as a respiratory rate (RR) less than or equal to 10 breaths per minute. Hypoxia was defined as an initial oxygen saturation (SaO2) less than or equal to 90%. Cardiovascular events were defined as cardiac arrest, need for cardiopulmonary resuscitation (CPR), elevated troponin and bradycardia of <50 bpm.

Individual sites were grouped into four regions: West (California, Oregon), Midwest (Colorado, Michigan, Missouri), Southeast (Georgia) and Northeast (New York, New Jersey, Pennsylvania). Ten sites contributed data between September 2020 and September 2023. One site in Missouri ended data collection in 2022. The Georgia and Colorado sites began data contribution in 2022.

### Toxicological analyses

Waste blood specimens were collected by site investigators and ToxIC staff. Serum samples drawn as part of routine clinical care were collected, de-identified and stored in at least 4°C until being sent to the Center for Forensic Science Research and Education (CFSRE) for analysis. Qualitative molecular identification consisted of liquid chromatography quadrupole time-of-flight mass spectrometry (LC-QTOF-MS) analysis with secondary analysis by liquid chromatography tandem quadrupole mass spectrometry (LC-QqQ-MS), when necessary. Current CFSRE toxicology testing contains over 1200 drugs, including therapeutics, traditional illicit drugs, novel psychoactive substances (NPSs), adulterants and other compounds. This methodology has been previously validated [20] and the molecular battery is frequently updated.

The illicit opioids of interest were fentanyl, fentanyl analogs (e.g. acetylfentanyl, carfentanil), nitazene analogs (e.g. isotonitazene, metonitazene) and other new synthetic opioids (e.g. brorphine), as well as previously prevalent synthetic opioids and heroin. The limit of detection for both xylazine and fentanyl was 0.1 ng/ml.

Biological samples were de-identified with a code linking the patient’s sample to the corresponding ToxIC clinical data entry by the site. Patients were categorized into those with xylazine detected (i.e. xylazine-positive group) and those without xylazine detected (i.-e. xylazine-negative controls) based on LC-QTOF-MS and/or LC-QQQ-MS.

### Data collection and management

Site-specific medical record data were abstracted and entered onto the secure, web-based Research Electronic Data Capture (REDCap) platform. Medical record data were de-identified and entered by trained medical toxicology physician site investigators and/or their research staff at each site. Data included age, gender identity, past medical and psychiatric comorbidities, suspected opioid name, treatment rendered (i.e. dose, amount and route of naloxone administration) and outcome, including presence or absence of organ system toxicity. Toxicological results were separately entered into REDCap and were blinded to clinical outcomes.

ToxIC data quality assurance is maintained by dedicated personnel in accordance with current best practices [21], including pilot testing prior to implementation, database logical checks, automated data cleaning, data tracking, secure encryption, procedure manuals and data abstractor training [22]. REDCap platform quality assurance confirmed that >90% of pertinent data fields were completed.

### Data analysis

Descriptive statistics are reported as medians with interquartile ranges and percentages. Categorical variables were evaluated using the chi-square test and Fisher’s exact test, when appropriate. Continuous variables were compared via Student’s *t*-test. Cohen’s *D* was computed for statistically significant bivariate associations to show effect sizes. All statistical assumptions were evaluated and verified.

Multivariable logistic regression analysis estimated the association between explanatory clinical variables (initial ED vital sign parameters) and study outcome (xylazine detection) when controlling for confounders. Data are reported as point estimates with corresponding 95% confidence intervals. Data analysis was performed on Stata/SE 16.1 (StataCorp LLC, College Station, TX, USA). The data analysis for the study was not pre-registered on a publicly available platform and results should be considered exploratory.

### Outcomes

To identify the clinical and demographic variables associated with xylazine in ED opioid overdose patients, xylazine detection in patient serum by LC-QTOF-MS was the primary outcome variable.

## RESULTS

**FIGURE 1:**
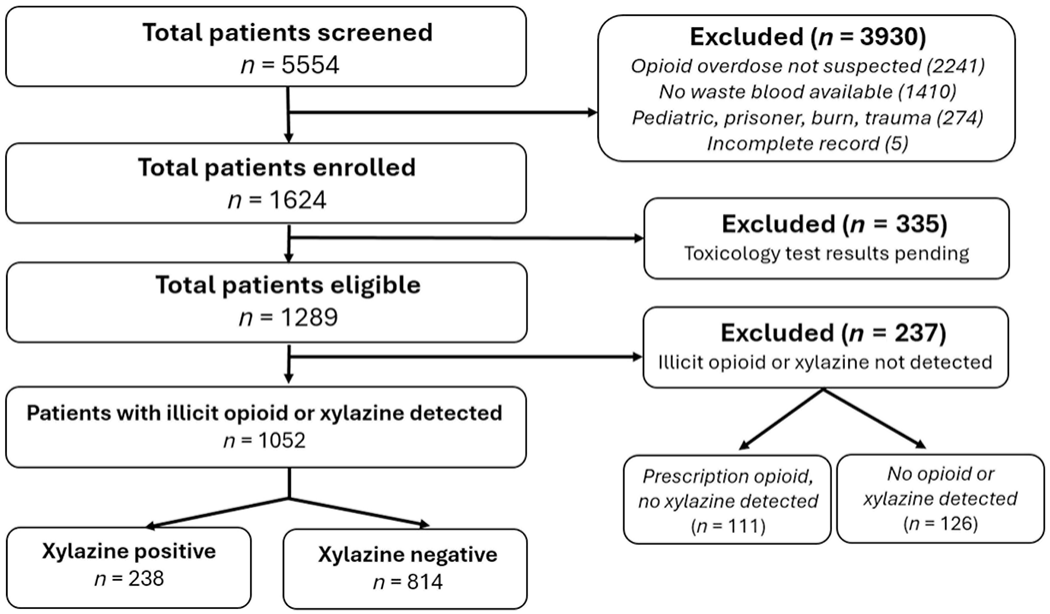
Patient eligibility and enrollment.

A total of 5554 patients were screened during the study period; of these, 1624 (29.2%) met the inclusion criteria for the Fentalog Study and 1289 (23.2%) had toxicology results completed at the time of analysis (Figure 1). Among those with results, 238 (22.6%) had xylazine detected and 814 (77.4%) had an illicit opioid without xylazine detected. The most common substances detected among those with results included fentanyl, stimulants and benzodiazepines (Table 1). Fentanyl was detected in 98.3% of patients with xylazine and in 97.2% of patients without xylazine. Stimulants and benzodiazepines were detected in 55.0% and 37.0% of patients with xylazine and in 58.9% and 28.4% of patients without xylazine, respectively. Heroin was detected in 15.1% of patients with xylazine and 7.6% of patients without xylazine. Xylazine without any opioid was detected in three patients (1.2%) and xylazine with a prescription opioid was detected in one patient (0.4%).

The demographic characteristics of the groups were similar (Table 1). Patients with xylazine were older, with a mean age of 44.8 years in the xylazine-positive group versus 41.5 years in the xylazine-negative group. Gender identity, race and ethnicity distributions were similar between the groups. Most (74.9%) patients with xylazine were in the Northeast region. Patients with xylazine had higher co-detection of benzodiazepines (37.0%) compared with those without xylazine (28.4%) (effect size, Cohen’s *D* = 0.19, *P* = 0.005). However, the magnitude of this difference was small. Both groups had similar co-detection of stimulants (55.0% xylazine positive, 58.8% xylazine negative, *P* = 0.29).

**FIGURE 2:**
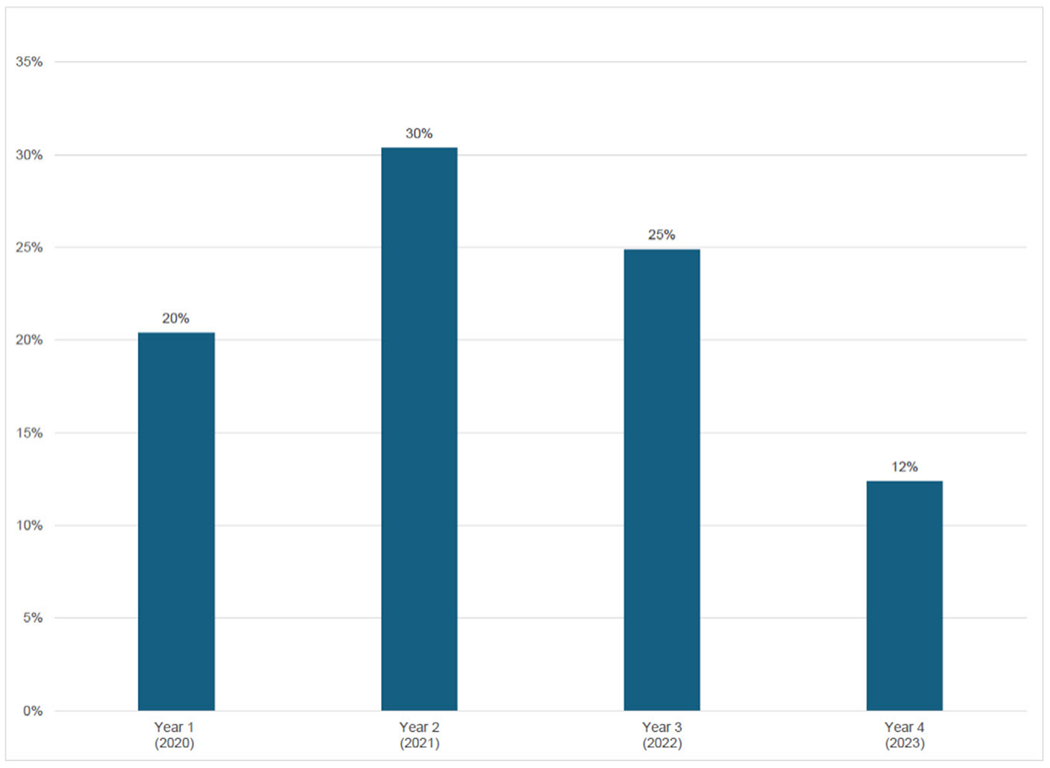
Overall prevalence of xylazine by year, 2020–2023.

Temporal data (Figure 2) show differences in xylazine detection over the study period. The highest frequency of xylazine detection occurred in year 2 (2021), while the lowest frequency of xylazine detection occurred in year 4 (2023) (*P* < 0.001). There was no increasing linear trend in xylazine detection across all years. Pairwise comparisons across years revealed a significant increase in xylazine detection in year 2 (2021) (*P* = 0.015) and a decline in xylazine detection in year 3 (2022) (*P* = 0.288) and year 4 (2023) (*P* = 0.022).

**FIGURE 3:**
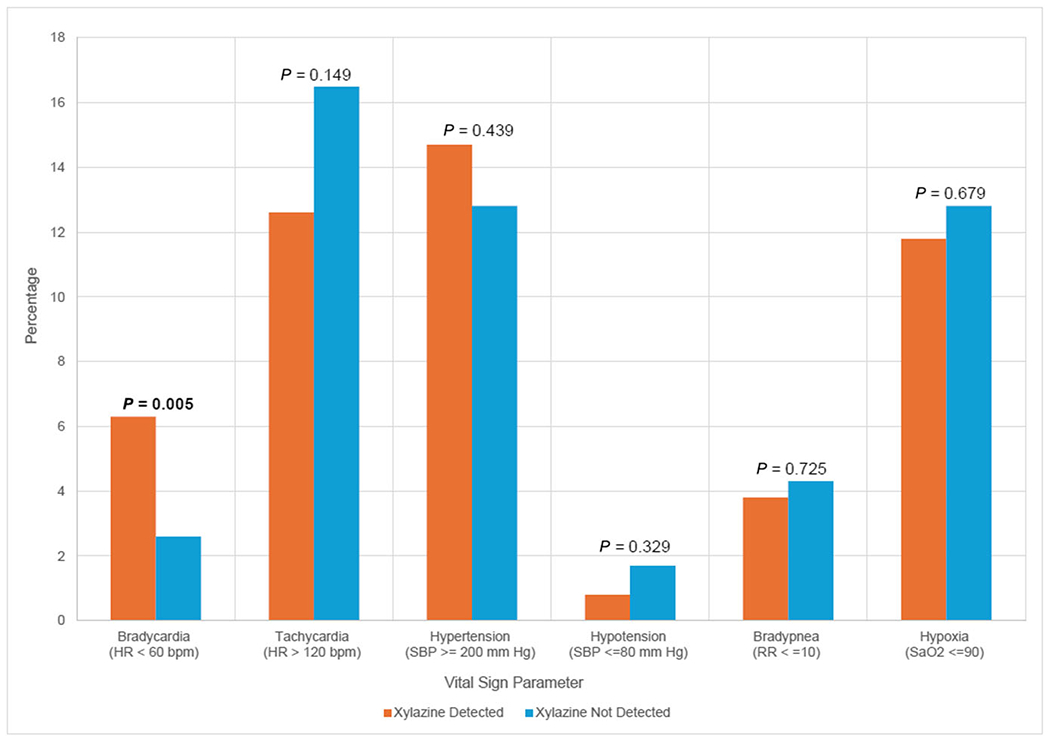
Vital sign predictors associated with xylazine detection. Abbreviations: HR = heart rate; SaO2 = oxygen saturation; SBP = systolic blood pressure.

Median initial ED vital sign parameters, including heart rate, systolic blood pressure and respiratory rates, were similar between groups. Median heart rate was 90.5 bpm (interquartile range, IQR = 77–105 bpm) in patients with xylazine and 96 bpm (IQR = 84–111 bpm) in patients without xylazine. Median systolic blood pressure was 130 mmHg (IQR = 114–145 mmHg) in patients with xylazine and 133 mmHg (IQR = 119–148 mmHg) in patients without xylazine. Median respiratory rate was 18 breaths per minute (IQR = 16–20 breaths per minute) in patients with xylazine and 18 breaths per minute (IQR = 15–20 breaths per minute) in patients without xylazine. Initial ED vital sign parameters were similar across groups except for frequency of bradycardia (Figure 3). Patients with xylazine had higher rates of bradycardia on initial ED vital signs (6.3%) compared with those without xylazine (2.6%). Similar frequencies of tachycardia (12.6% vs 16.4%), hypertension (14.7% vs 12.8%), hypotension (0.8% vs 1.7%), bradypnea (3.8% vs 4.3%) and hypoxia (11.7% vs 12.8%) were noted for patients with xylazine and patients without xylazine (Figure 3).

Most patients in both groups (74.7% xylazine positive, 79.6% xylazine negative) received naloxone and the median naloxone bolus dose in both groups was 2.0 mg. Patients with xylazine had a lower median total naloxone dose compared with those without xylazine (3.75 mg vs 6.88 mg).

Cardiovascular events were less frequent in patients with xylazine compared with those without xylazine (Table 2). No cardiovascular events within 4 hours of ED arrival were reported in 91.2% of patients with xylazine, while 85.6% of patients without xylazine had no cardiovascular events (*P* = 0.03). Similarly, patients with xylazine received cardiopulmonary resuscitation less often (4.6% vs 12.8%, *P* = 0.01). Pulmonary events were also less frequent in patients with xylazine (82.7% vs 76.9% with no pulmonary events detected in the first 4 hours of arrival, *P* = 0.05). When pulmonary events occurred, including intubation, higher rates occurred in patients testing xylazine negative (5.0% vs 8.9%, *P* = 0.06).

Mortality did not significantly differ between the two groups (Table 2). Patients with xylazine detected had a lower frequency of mortality (0.4% vs 2.1%), but this difference was not statistically significant (*P* = 0.08). Intensive care unit (ICU) disposition from the ED significantly differed between the two groups: 17.4% of patients with opioids alone were admitted to the ICU compared with 10.9% of patients with xylazine detected (*P* = 0.02).

After adjusting for age, race/ethnicity, gender identity and psychiatric history, multifactorial logistic regression modeling demonstrated several positive predictors of xylazine detection in serum sampling of patients attending an ED with opioid overdose (Table 3). Factors associated with higher odds of xylazine detection in patients with opioid overdose included bradycardia on initial ED vital signs (adjusted odds ratio, aOR = 2.26, 95% CI = 1.14–4.50) and age 60+ years (aOR = 2.33, 95% CI = 1.15–4.72). Compared with the Northeast region, non-Northeast regions of the USA (Central aOR = 0.60, 95% CI = 0.40–0.88; Southeast aOR = 0.03, 95% CI = 0.01–0.10; West aOR = 0.30, 95% CI = 0.12–0.75) were associated with a lower odds of xylazine detection. Other factors associated with lower odds of xylazine detection included Hispanic ethnicity (aOR = 0.62, 95% CI = 0.40–0.98) and tachycardic heart rate on initial ED vital signs (aOR = 0.62, 95% CI = 0.40–0.98). Model goodness-of-fit testing revealed a *P*-value of 0.9723, which indicates a good fit for the model.

## DISCUSSION

In this multi-center study of patients attending an ED with opioid overdose, we found that US region and bradycardia on ED arrival were positive predictors of xylazine detection. This study is the first to describe clinical parameters associated with xylazine co-detection in ED patients with non-fatal opioid overdose. Differences in clinical parameters were small and limited in their ability to fully characterize a xylazine toxidrome but provide a foundation for understanding the clinical effects of xylazine in acute opioid overdose.

Recent studies have described characteristics, including higher opioid quantities, housing instability, polysubstance use and hepatitis C infection, associated with xylazine detection but are limited to post-mortem patients [17] or survey studies [10, 19]. Our population is unique because it represents *non-fatal* overdose patients and better characterizes xylazine co-detection among a larger group of individuals with non-prescription opioid use. By incorporating clinically relevant variables, such as vital signs, our findings aid clinicians in identifying patients who may be intentionally or unintentionally exposed to xylazine and aligning ED and harm reduction resources with their clinical care.

Our finding that US Northeast regions had higher odds of xylazine detection aligns with current epidemiologic trends. Other states reporting xylazine detection have included Connecticut, Pennsylvania and Maryland. National trends of drug seizure samples and overdose deaths note the highest reporting prevalence in Northeast states [23]. However, xylazine co-detection with opioids is increasing in other US regions, including the Southeast and Southwest. Temporal trends in xylazine detection also exist. We found a significant increase in xylazine detection in 2021, with a subsequent decrease in detection in later study years. Temporal trends are common among drug supply adulterants, with new and old adulterants cycling through the illicit drug supply. Still, xylazine has not been eliminated from illicit opioid supply and was detected at 12% frequency in 2023. Robust, multi-source toxicosurveillance systems, such as the ToxIC Fentalog Study, are a critical tool to monitor both regional and temporal trends in the emergence and growth of novel substances.

Bradycardia on initial ED vital signs was associated with xylazine detection in non-fatal opioid overdose. This finding is important in helping characterize the clinical toxidrome of patients exposed to xylazine-adulterated opioids. Bradycardia is a well-described clinical sign associated with xylazine toxicity in single-agent overdose [24–26]. The mechanism of action of xylazine as an alpha-2 agonist contributes to bradycardia by blocking post-synaptic receptors from norepinephrine uptake. Norepinephrine is a neurotransmitter with both excitatory and inhibitory effects. Blocking downstream norepinephrine uptake results in physiologic signs, including bradycardia and hypotension. Given this mechanism of action, we hypothesized that xylazine detection would be associated with bradycardia and hypotension on initial ED vital signs. However, only bradycardia was significantly associated with xylazine detection in this population, and hypotension was not significantly associated.

Isolated bradycardia in non-fatal xylazine–fentanyl overdose may arise through several mechanisms. One mechanism may be associated with xylazine and non-specific alpha-2 receptor binding. Located in the CNS and peripheral vasculature, alpha-2 receptors have different mechanisms of action. CNS alpha-2 receptor activation decreases sympathetic outflow and sympathetic tone, resulting in bradycardia and hypotension [27]. Pre-synaptic alpha-2 receptors activate negative feedback and decrease synaptic norepinephrine release. Alpha-2 receptor activation on peripheral vasculature results in vasoconstriction and reflex bradycardia. Therefore, isolated bradycardia without hypotension may be related to a lack of xylazine alpha-2 receptor specificity and subsequent peripheral and central alpha-2 receptor binding effects.

The combination of the alpha effects of xylazine and hypoxia from fentanyl use could also contribute to bradycardia. Fentanyl overdose can cause severe respiratory depression and hypoxia, leading to hypoxia-induced bradycardia. Synergism between hypoxia-induced bradycardia from fentanyl overdose and bradycardia from xylazine alpha-2 binding may explain our findings. Of note, there was no significant difference in initial oxygen saturation between the two groups. However, bradycardia induced by multiple physiologic mechanisms related to xylazine–fentanyl non-fatal overdose should be explored.

A recent National Institute on Drug Abuse (NIDA) study exploring the physiologic effects of xylazine-adulterated fentanyl and heroin on rats revealed that adding xylazine to opioids delayed brain rebound from cerebral hypoxia [28]. Given our findings of bradycardia associated with xylazine-adulterated fentanyl, one hypothesis for prolonged cerebral hypoxia may be prolonged bradycardia and subsequent brain hypoperfusion from xylazine.

Our finding that male patients had a higher odds of xylazine detection, while not statistically significant, is interesting. Animal models suggest sex differences in withdrawal patterns [29]. Female rats demonstrate greater withdrawal behaviors when exposed to fentanyl–xylazine and administered naloxone or atipamezole, a veterinary alpha-antagonist [29]. Our findings could indicate sex differences in physiologic responses in xylazine-adulterated fentanyl overdose and withdrawal. Future work may explore differences in xylazine-adulterated fentanyl withdrawal physiology and symptoms to characterize health outcomes and use patterns for male and female patients.

Our models describe an association between older patients (aged 60+ years) and increased odds of xylazine detection. Patients aged 60+ years or older comprised approximately 20% of the sample and patient ages were similarly distributed across xylazine-positive and --negative groups. This association may be related to xylazine pharmacokinetics in older patients. No published data exist on xylazine pharmacokinetics in humans, but animal models support xylazine hepatic metabolism via cytochrome P450 (CYP P450) enzymes and renal excretion. Xylazine detection in older adults may be explained by co-existing renal or liver disease or competing CYP metabolism from other substances or medications. Older patients may seek out xylazine-adulterated opioids for physiologic effects, such as CNS sedation, or may have different frequency-of-use patterns. Future work should explore age-related differences in attitudes and sentiments around xylazine-adulterated fentanyl.

Finally, we found no difference in mortality between patients with or without xylazine detected, but a lower odds of ICU admission among those with xylazine detected. The lack of a difference in mortality rates is expected, because the overall mortality rate is low and did not have sufficient power to detect a significant difference. The lower odds of ICU admission among those with xylazine detected is interesting and warrants future investigation. Previous work found that patients with xylazine detected had less severe clinical outcomes in opioid overdose, such as cardiac arrest and coma [20]. Similarly, the lower odds of ICU admission may be indicative of less severe overdose or less total fentanyl exposure among patients attending an ED with opioid overdose. Future work should explore associations between hospital and treatment outcomes and the presence of xylazine and other opioid adulterants.

Our study findings contribute novel data to the growing medical literature and research agenda on the clinical health effects of xylazine for patients with opioid use disorder [5, 20]. These findings bolster the growing inventory of xylazine-associated research questions that require timely, multi-modal, data-driven approaches to understand the evolving epidemiology and health effects of xylazine-adulterated fentanyl. As discussed above and in expert research agendas, potential areas for new discovery in xylazine-related research include the assessment of xylazine pharmacokinetics and metabolism, and the qualitative analysis of xylazine-adulterated fentanyl use patterns [5].

### Limitations

This study has several relevant limitations. Among the patients screened, 25.4% lacked routine laboratory testing and waste clinical specimens were not available. Blood sampling provided only qualitative detection of illicit opioids, xylazine and other novel substances. Quantitative serum concentrations were not measured and only associations between xylazine detection and relevant clinical predictors can be inferred. Data on the timing of substance use relative to ED arrival were not collected, and the window of detection for serum xylazine is unknown. Substances detected on serum sampling may represent prior exposure and not the non-fatal overdose exposure associated with the ED visit. The absence of xylazine or other substances may reflect rapid metabolism or serum clearance, and subsequent short physiologic detection time. The study was limited to opioid overdoses requiring ED transport; pre-hospital fatalities and associated clinical predictors were not assessed. Finally, participating sites were limited to US urban areas, and the findings may not be applicable to rural regions.

## CONCLUSION

In this multi-center study of ED patients with non-fatal opioid overdose and xylazine, we found that bradycardia on initial vital signs was associated with an increased likelihood of xylazine detection on serum testing. These novel findings help in starting to characterize the clinical toxicity associated with xylazine-adulterated fentanyl. Xylazine continues to have higher detection rates among patients in Northeast regions of the USA. Study findings also highlight subpopulations (i.e. older adults, females) who may have different opioid use patterns owing to xylazine adulteration.

## Figures and Tables

**TABLE 1:** Demographic characteristics of patients with and without xylazine detected.

	Xylazine detected *n* = 238, 22.6%	%	Xylazine not detected *n* = 814, 77.4%	%	P
**Mean age (years)**	44.8		41.5		
**Age (years, %)**					
18–25	14	14.0	86	96.0	0.014
26–35	72	20.9	273	79.1	
36–45	54	23.9	172	76.1	
46–59	46	21.4	169	78.6	
60+	52	31.3	114	68.7	
**Gender identity (%)**					
Female	52	18.7	226	81.3	0.094
Male	185	24.2	578	75.6	
Transgender/non-binary/other	1	9.1	10	90.9	
**Race (%)**					
White	113	72.4	43	27.6	<0.00001
Black	70	24.0	222	76.0	
Other	55	25.7	159	74.3	
**Ethnicity (%)**					
Hispanic	42	18.7	183	81.3	
**Region (%)**					
Northeast	178	31.4	388	68.6	<0.00001
Midwest	51	23.9	162	76.1	
Southeast	3	1.4	219	98.6	
West Coast	6	11.8	45	88.2	
**Psychiatric history (%)**					
No psychiatric history	69	27.2	185	72.8	0.19
Any	85	18.8	368	81.2	
Anxiety	9	21.4	33	78.6	
Bipolar disorder	14	25.9	40	74.1	
Depression	37	23.1	123	76.9	
Post-traumatic stress disorder (PTSD)	8	22.9	27	77.1	
Schizophrenia	16	29.6	38	70.4	
**Substances detected (%)**					
Fentanyl and fentanyl analogs	234	22.8	791	77.2	0.00039
Heroin	36	36.7	62	63.2	
Other non-fentanyl illicit opioids	41	34.5	78	65.5	
Benzodiazepines	88	27.6	231	72.4	
Stimulants	131	21.5	479	78.5	

**TABLE 2:** Clinical events and outcomes among patients with and without xylazine detection.

	Xylazine detected *n* = 238 (22.6%)	%	Xylazine not detected *n* = 814 (77.4%)	%	P
**Cardiovascular events**					
No cardiovascular events within 4 h	217	91.2	698	85.8	0.029
Received CPR	11	4.6	83	10.2	0.008
**CNS events**					
No CNS events within 4 h	93	39.1	343	42.1	0.399
Coma within 4 h	104	43.7	342	42.0	0.644
Agitation within 4 h	44	18.5	126	15.5	0.267
**Pulmonary events**					
No pulmonary events within 4 h	197	82.8	626	76.9	0.054
Non-invasive positive pressure within 4 h (CPAP/BIPAP)	4	1.7	33	4.1	0.08
Intubated within 4 h	12	5.0	72	8.9	0.057
**Overall outcomes**					
Discharged from the ED	158	66.4	456	56.0	0.182
Admitted to hospital	61	25.6	245	30.1	0.182
ICU admission	26	10.9	142	17.4	0.016
Death	1	0.4	17	2.1	0.081

*Note*: Abbreviations: BIPAP = bilevel positive airway pressure; CNS = central nervous system; CPAP = continuous positive airway pressure; CPR = cardiopulmonary resuscitation; ED = emergency department; ICU = intensive care unit.

**TABLE 3:** Predictors of xylazine detection.

Variable name	aOR	95% CI
**Year**		
Year 1 (2020)	Ref.	Ref.
**Year 2 (2021)**	**2.02**	**1.14–3.56**
Year 3 (2022)	1.64	0.96–2.79
Year 4 (2023)	1.07	0.56–2.07
**Age category**		
18–25 years	Ref.	Ref.
26–35 years	1.71	0.88–3.31
36–45 years	1.79	0.90–3.56
46–59 years	1.57	0.78–3.17
**60 + years**	**2.33**	**1.15–4.72**
**Gender identity**		
Female	Ref.	Ref.
Male	1.44	0.99–2.08
**Race**		
White	Ref.	Ref.
Black	0.73	0.50–1.08
Other	1.11	0.71–1.75
**Ethnicity**		
Non-Hispanic	Ref.	Ref.
**Hispanic**	**0.62**	**0.40–0.98**
**Region**		
Northeast	Ref.	Ref.
**Midwest**	**0.60**	**0.40–0.88**
**Southeast**	**0.03**	**0.01–0.10**
**West Coast**	**0.30**	**0.12–0.75**
**Initial ED vital signs**		
Tachycardia	0.62	0.39–0.98
**Bradycardia (<60 bpm)**	**2.26**	**1.14–4.50**
**Other variables**		
History of depression	1.07	0.71–1.59
Agitation within 4 h	1.30	0.86–1.96
No response to first naloxone dose	0.76	0.49–1.19

*Note*: Model goodness of fit: prob > χ^2^ = 0.9723. Values in **bold** indicate statistical significance. ED = emergency department.

## Data Availability

The data that support the findings of this study are available from the corresponding author upon reasonable request.
